# Analysis of the impact of social insurance on farmers in China: A study exploring subjective perceptions of well-being and the mechanisms of common prosperity

**DOI:** 10.3389/fpsyg.2022.1004581

**Published:** 2022-12-08

**Authors:** Yongsheng Cheng, Deyuan Zhang

**Affiliations:** ^1^School of Business, Fuyang Normal University, Fuyang, Anhui, China; ^2^Academy of Strategies for Innovation and Development, Anhui University, Hefei, China

**Keywords:** new rural social pension insurance, rural residents, subjective well-being, mediation effect, CFPS

## Abstract

**Objective:**

Exploring common prosperity in China, this study focuses on the impact of social pension insurance on the well-being of rural communities. It explores the direct beneficiaries and policy effects of the Rural Social Pension Insurance system (RSPI), which was piloted in 2009 and achieved full coverage in 2012. It summarizes the performance and implementation of social pension insurance and the development of the rural social pension system.

**Methods:**

The article uses microdata from the four most recent periods of China Family Panel Studies (CFPS), which were undertaken in 2012, 2014, 2016, and 2018, and uses the Order Probit model to analyze the effects of participating in insurance on rural residents in terms of their subjective well-being. The study identifies diverse effects on farmers from different age groups, genders, and regions, with a focus on subjective well-being. The article also tests the mediating effects of health status and self-rated social status on farmers’ subjective well-being and their mechanisms of action.

**Results:**

Findings reveal that participating in the insurance system significantly improved the subjective well-being of rural residents. Its biggest beneficiaries were groups of rural residents with poor health status, living in good overall conditions. Taking into account the most recent aims of this policy, to promote rural revitalization and common prosperity, further optimization of the rural pension insurance system should improve the living standards of low-income groups, enabling more comprehensive coverage, and potentially helping to mitigate the risk of returning to poverty due to illness.

**Conclusion:**

Developments in basic social security and the rural basic pension system could effectively guarantee the basic standards of living of rural residents. Future development of the system should take into account the heterogeneous characteristics of rural residents and implement social pension protection policies in accordance with local conditions.

## 1. Introduction

As an important feature of Chinese modernization, common prosperity is an essential requirement of socialism, encompassing the common pursuit of “double prosperity” in terms of both the material and spiritual life of all people (The Communist Party Member Network, 2022). The creation of a “happy China” through common prosperity has become an important priority in recent years and the country has entered a new era of socialist modernization and facilitating a move to people being more comprehensively well-off in terms of common prosperity. This also coincides with the value orientation of happiness economics, outlining that happiness is the goal of residents and fits the value expectations of government policies. This is a universal expectation and an ultimate goal in human life all over the world.

Since R. Easterlin introduced happiness into economics research in the 1970s. Since then, it has attracted widespread academic attention because of its strong subjective focus and its methods of measuring individual well-being more comprehensively (Easterlin, 1995), and a focus on subjective well-being has become the mainstay of happiness research (Easterlin et al., 2012). Subjective well-being as “happiness” is the most important aspect of happiness research. As an important object of study in “happiness economics,” subjective well-being reflects not only the material aspects of people’s daily life but also their spiritual satisfaction. This involves measuring individual utility and national welfare and is a comprehensive indicator through which we can evaluate people’s life satisfaction and well-being (Ferrer and Carbonell, 2005; Oshio et al., 2011). According to the Global Happiness Report 2022, although China’s happiness has improved, it is still 2.24% lower than that of Finland, which has been in first place for many years. Since 2000, the size and proportion of China’s elderly population have been increasing year by year, with 267 million people aged 60 and above (18.9%), and 201 million people aged 65 and above (14.2%) at the end of 2021. It is expected that by the middle of the 21st century, China will enter a stage of deep aging and that the size of the elderly population will reach about 500 million people. The proportion of people aged 65 and above in rural areas will be as high as 46.4%, which is approximately 2.1 times that of urban areas. As a society, China will face increased pressures due to the old age of urban residents (Wang et al., 2019) and the growth dilemma of “happiness stagnation” will be more complicated and severe in rural residents.

In response to the pressures of these aging populations, the Chinese government has introduced a series of policies that attempt to address growing rural pension problems, including the Rural Social Pension Insurance system (RSPI), which launched as a pilot program in 2009 and achieved full coverage in 2012. As one of the core elements of China’s current rural social pension insurance system, the 2012 RSPI is still one of the key initiatives for coping with the aging rural population and improving the future living conditions of elderly people in rural areas. The 2012 RSPI is also an important institutional guarantee that the government aims to improve the quality of life of rural residents and ensure a balanced urban and rural pension service. It has become a safety net and social shock absorber for the wider population, providing bottom-up basic protection for the public. On the theoretical level, the policy design of the 2012 RSPI aims to provide a system that will help guarantee China’s aim to eradicate poverty and build a moderately prosperous society. The most important feature of this aim adopts a model that combines three funding channels: including individual contributions, collective subsidies, and government subsidies, and providing a new pension income for people in agricultural households that will relieve pressures on living and improve their living standards through external subsidized support from the government. The government provides this new pension income for people in agricultural households to relieve their living pressures, improve their standard of living, provide some support and protection for economic life, and improve their quality of life (Yi and Zhao, 2021). As a compensatory social pension system, the 2012 RSPI policy provides farmers with stable material security through regular pension payments and increases their income level in old age. In addition, for the rural elderly, the 2012 RSPI policy also has a greater effect in securing basic livelihoods, as pensions increase the total wealth of the elderly and this increase in total wealth enables the rural elderly to reduce the various types of agricultural and non-agricultural labor they supply or withdraw from the labor market, facilitating their ability to enjoy leisure time, and thus improving their physical functional status and ability to care for themselves (Liu et al., 2021).

Despite these aims, by the end of 2021, China’s basic pension insurance participation rate was approximately 72.82%, which is far from the ultimate goal of “universal coverage and comprehensive coverage.” Since it has now been over 10 years since the implementation of the 2012 RSPI, the question of how the effectiveness of this policy can be assessed needs to be addressed. Has the social pension insurance system, which aims to equalize basic public services, achieved the expected goal of providing rural residents with a sense of security in their old age? The answer to this question is ultimately reflected in these systems have improved the living conditions of rural residents. Based upon the fact that the system is designed to provide “wide coverage” and contribute “more contributions, more benefits,” has the 2012 RSPI changed the subjective sense of well-being of rural residents, and how will it do so in the future? Will it alleviate the plight of rural residents and improve their quality of life? Which groups of rural residents will benefit most? Scientific and rigorous measurement studies that address these questions are thus of great practical significance for the formulation and optimization of public policies in this new era.

## 2. Policy background and literature review

In the face of the increasingly serious problem of an aging population, gradually promoting multi-level pension security system and continuously improving social security policies are important fundamental solutions. From a historical perspective, the security systems that support China’s older rural populations can be roughly divided into four stages. First, the germination and incubation period between 1954 and 1985: this stage saw the implementation of the “five security support” and collective pension system (Wang, 2019), focusing on the loss of income sources and living without support for specific groups, such as farmers and veterans, aiming to provide appropriate production and living security and supporting the costs associated with living, sickness, death, and burial, etc. This created a precedent for China’s rural social pensions and opened up the exploration of a pension insurance system. Second, the initial exploration period between 1986 and 1998, during which rural social pension insurance was implemented. This system mainly relied on farmers’ unilateral contributions, proving a social security model for rural residents that enabled them to implement self-saving, generally known as “old farmers’ insurance.” However, due to a lack of government investment and farmers’ unwillingness to participate in insurance, the system was withdrawn in 1998 when the state rectified the insurance industry. Third, the period of exploration and adjustment between 1999 and 2008. This phase of rural social pension insurance was mainly focused on the independent exploration of provinces and formed some typical experiences and practices, but at the national level is still in the policy gap period. The fourth period is that of steady development from 2009 to the present, a time marked by the State Council’s promulgation and implementation of the “Guidance Opinions on the Launching of New Rural Social Pension Insurance Pilot,” which officially opened a new chapter in the development of institutional pension security in rural areas. In 2011, the State Council issued and implemented the “Guidance Opinions on the Launching of Urban Residents’ Social Pension Insurance Pilot.” In July 2014, the 2012 RSPI system was officially recognized and merged with the urban residential insurance system, and a national basic pension insurance system was established.

In literature on these stages, attention to social insurance is largely focused on macro and micro aspects. The macro aspects are mostly explained in terms of institutional design (Wang and Hetzler, 2015; Caro and Parada-Contzen, 2022) and institutional effectiveness (He and Jiang, 2015; Zhang et al., 2016; Huang and Zhang, 2021). Many studies have found that the predictable results derived from these traditional theories are not consistent with the real evidence. In recent years, more relevant studies on micro aspects have focused on influencing factors and group differences levels, and literature mainly reflects on the relationship between higher income and happiness enhancement (Zhao et al., 2013) and the relationship between individual characteristics and happiness (Mackerron, 2012; Zhou et al., 2022). The conclusions of these different studies are not identical (Liu et al., 2013) and few studies have focused in depth on the mechanisms of influence and the pathways of action.

To date, little attention has been paid to the relationship between rural social pension insurance and subjective well-being in general, and theoretical research has to an extent, lagged far behind the development of practice. Using 2-period CHARLS panel data, He and Zhou (2016) confirmed that the new rural insurance can effectively improve the degree of depression of rural residents; however, the same use of CHARLS data from the Gansu and Zhejiang provinces in 2008 and 2012 led to the opposite conclusion, arguing that family retirement is still the current mainstream concept of retirement in rural areas, coupled with a lack of external subsidies, meaning it is difficult to increase the welfare of the rural elderly (Xue, 2012; Xie, 2015; Cheng and Hua, 2020). In addition, the scope of 2012 RSPI coverage does not only involve a group of residents over 60 years old but also has an impact on rural residents under 60 years old, while the above-mentioned studies do not pay enough attention to heterogeneity, the in-depth research on the relationship between the social pension insurance and subjective welfare is very limited, which provides room for expansion of this manuscript. Specifically, this manuscript attempts to improve the existing studies in the following three aspects, hoping to make up for the “shortcomings” and regrets of the above-mentioned studies, which are also possible contributions of this study. First, in terms of research data, the total amount of sample data is sufficient and covers a wide range, which can better reflect the current situation of rural residents and help ensure that the research results are representative. Second, in terms of research methodology, can participation in the 2012 RSPI enhance farmers’ subjective perception of happiness? To date, existing studies do not provide a convincing answer. Using the latest four periods of large-volume CFPS data from 2012, 2014, 2016, and 2018, the present study applies scientific and rigorous econometric strategies to clarify the magnitude and paths of the effect of participation on rural residents, to provide systematic empirical data that enables us to measure the impact of old-age security at an institutional level. The empirical research methods used in the present study differ from those used in previous research by seeking more consistent and comparable analysis through matching methods. This empirical research methodology innovates by seeking more consistent and comparable samples, to avoid the possible shortcomings brought about by the self selection bias of survey respondents. Third, in terms of research significance, previous studies have mainly focused on urban and rural residents or special groups, such as migrant workers and the elderly (Deng and Yang, 2019; Zhao and Feng, 2020), but few have focused on rural residents alone. Moreover, the introduction of public policy variables into the factors influencing subjective well-being is conducive to providing theoretical insights into policies that might help society to cope with increasing aging, the construction of rural social security systems, and help accelerate the realization of common prosperity.

The research structure design and the overall ideas of the article are arranged as follows: in the introduction, the background is explained and the origin of the problem is presented. The policy background and literature review are then discussed in the second section, which briefly explains the policy background of social insurance, comprehensively comparing existing literature and briefly reviewing the shortcomings of these studies before presenting the fundamental ideas and possible innovations of this article. The third section undertakes a theoretical analysis and presents the research hypothesis, which examines the relationship between social insurance, subjective well-being, and the possible mechanisms of action involved, constructing a theoretical hypothesis model of social insurance and subjective well-being. The fourth part of the article then explores the data source, variable selection, and model selection, and defines the relevant variables and the basis of their selection according to the specification. Section five presents the article’s empirical results and undertakes analysis based on OLS benchmark regression. This section also undertakes further in-depth estimation by the Order Probit model, with an extended analysis of the individual effect test and a discussion of endogeneity using the Hausman test and PSM estimation. The sixth part tests the mechanism of relevant effects, which are further verified by the mediation model and Sobel test. Finally, the seventh section presents research conclusions and countermeasures.

## 3. Theoretical analysis and research hypothesis

Psychology believes that individual well-being broadly encompasses three dimensions that are connected to the innate genetic factors acquired by environmental influences and individual psychological traits. These include the influence of family environment, individual characteristics, social capital, and other extrinsic factors that might affect well-being (Martin, 2010). Although happiness economics also considers it to be a subjective feeling that is determined by factors such as one’s thoughts, mood, and state of mind, it focuses more on portraying a personal utility. When individual capital and external environmental factors change, people make certain decisions to maximize individual utility to enhance their happiness level. Neoclassical labor supply theory summarizes the utility function as the choices made by workers based on leisure and consumption under the condition of individual utility maximization. In terms of the theory of leisure economy, there is also a certain complementary effect between leisure time and working time (Wei and Lü, 2018). To further simplify the relationship presentation, the study assumes that individual lifetime time T contains only labor time and leisure time, *T_i_* is individual labor time, individual leisure time is T−*T*_*i*_, and tries to construct individual utility functions of the following form (Chen et al., 2020):


(1) $$\text{E}=\text{E }\left(\left(T-T_{i}\right)\;,\text{Y}\right)$$

$$s.t.Y=R\;\left(T_{i},\;Z\right)+X+O$$

In the above Equation 1, E is individual utility, which depends on individual leisure time T−*T*_*i*_and individual consumption Y; *R* (*T*_*i*_,*Z*) is total labor income, *X* is pension input, *Z* is capital input, and *O* is other transfer income. Under the realistic budget constraint, the individual utility of insured farmers and their subjective well-being may be enhanced because the pension income from social insurance reduces labor intensity and enhances leisure enjoyment. It may also enhance their subjective utility because the pension brings an increase in disposable income (Zhang and Tan, 2018), relaxes budget constraints, and increases consumption levels (Hong, 2017; Yue and You, 2018). Since subjective well-being originates from individuals’ positive cognitive and affective assessment of their own living conditions, there must also be distinct individual heterogeneity characteristics (Huang et al., 2016; Leng et al., 2022). Accordingly, the current study proposes the following research hypothesis:

H_1_: rural residents’ participation in social insurance can positively affect their individual subjective well-being, which is the main presentation of the social insurance happiness effect.

H_2_: the impact of social insurance on rural residents’ subjective well-being may be heterogeneous, with different insured individuals showing different perceptions of subjective well-being.

As a socialized livelihood project with economic welfare, social insurance is directly related to people’s well-being, and maintaining health should be one of the basic objectives of the system design (Ni, 2020). As a form of government redistribution, it is supposed to play an important role in improving people’s health status (He and Li, 2019). However, established studies have generally focused on health status as a control variable, arguing that social insurance, despite its short implementation time and low level of coverage, still improves the quality of old age and enhances the physical health of rural elderly people to some extent (Bai and Gu, 2019; Chen et al., 2019) and that it can reduce the “no care for the elderly” probability, providing a level of survival guarantee for subsequent life and significantly reducing the risk of long-term multidimensional health poverty (Galiani et al., 2014) and reducing the degree of happiness “deprivation” because rural insurance can eliminate the residents’ worries about their old age (Oswald and Powdthavee, 2008; Hou, 2018). It can also reduce the dependence of elderly residents on their children, reduce their psychological stress and improve their self-esteem (Wang et al., 2004), making their evaluation of their health more optimistic. At the same time, studies have reached different conclusions, finding that the effect of pension insurance on the physical health of the elderly is limited (Xiang and Yao, 2016), because the act of participating in insurance exposes the rural elderly to severe consumption deprivation, leading to a deeper degree of consumption disparity between individuals, which in turn may create a sense of relative deprivation and have an impact on the nutrition and health of the rural elderly. This, in turn, impairs personal physical and mental health (Huang et al., 2019), and indirectly affects the well-being of the elderly. In this regard, this manuscript proposes another research hypothesis 3:

H_3_: self-rated health status may play a mediating role in the relationship between social insurance and well-being, because social insurance may affect rural residents’ self-rated health level, which in turn affects residents’ well-being through self-rated health status.

The pursuit of social status is also a basic human motivation (Chen et al., 2018). According to the theory of relative deprivation, individuals with lower levels of status are often at a disadvantage compared with “others” and are more likely to have negative feelings toward the outside world (Long and Liang, 2019; Pu and Zhao, 2020). Rural areas in China are influenced by the traditional culture of “not having to worry about scarcity but not having to worry about inequality,” rural residents’ value judgments are embedded in the acquaintance society based on blood and geographical ties. People from these areas tend to externally present themselves by comparing their welfare with those around them and judging whether they are at a disadvantage. In this context, uninsured farmers may have a sense of relative deprivation that “we are entitled to but actually excluded from” in the process of social comparison, thus generating negative emotions. In this situation, the positive mediating effect of self-assessed social status is more significant (Zhang and Sun, 2019; Zhang and Qin, 2020). A person’s social status depends on wealth and income (economic status), power, and prestige. It also determines the degree of respect, income distribution, opportunities, personal talent, and self-actualization, which is in line with Maslow’s hierarchical needs theory for belonging, respect, and self-actualization (Xu and Chen, 2017; Ma et al., 2018; Hou and Ge, 2020). Accordingly, this manuscript proposes research hypothesis 4:

H_4_: self-rated social status may play a fully mediating role between social insurance and subjective well-being, as part of which social insurance influences residents’ well-being through self-rated social status.

**FIGURE 1:**
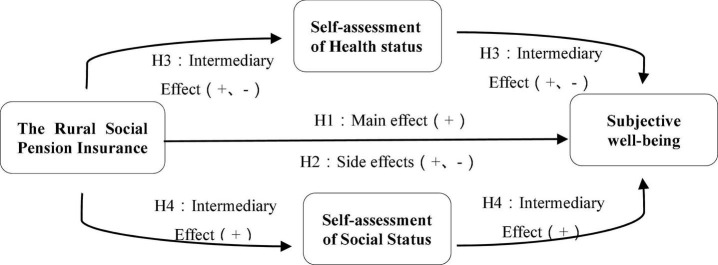
Theoretical hypothesis model of social insurance and subjective well-being.

Based on the above theoretical analysis, this manuscript constructs a theoretical hypothesis model of social insurance and subjective well-being of rural residents, as shown in Figure 1, incorporating self-rated health status and self-rated social status into a unified analytical framework to verify the mechanism of social insurance influence on farmers’ subjective well-being through self-rated health status and self-rated social status.

## 4. Data and method

### 4.1 Data

This manuscript is based on data released by the China Family Panel Studies (CFPS) conducted by Peking University (Xie and Hu, 2014). The CFPS survey is methodologically rigorous and representative, covering 16,000 households in 635 villages (communities) in 162 counties in 25 provinces, municipalities, and autonomous regions. Its stratified multi-stage systematic probability sampling approach improves the efficiency of the sample to represent the whole population, covering approximately 95% of the Chinese population (Xie and Lu, 2015). CFPS research has wide coverage, high authority, and large sample information, with good reliability and validity. It is a nationally representative sample, with complete public data currently available as of 2018. To ensure the scientific scope of this study and the comparability of data, the article used a valid sample of 43,700 after eliminating some missing samples of variables, including 749 samples in 2012, 13,918 samples in 2014, 15,793 samples in 2016, 13,240 samples in 2018, and 11,894 respondents in the sample participated in four surveys at the same time, according to the non-balanced short panel data processed.

### 4.2 Variable selection and sample description

According to the needs of the study, the study selected a sample of adults whose current household status was marked as agricultural in the questionnaire, with the youngest resident in the sample being 16 years old (Table 1).^1^

**TABLE 1:** Variable definition table (*N* = 43,700).

Variable type	Variable name	Variable symbols	Variable definition
Explained variables	Subjective well-being perception	Happiness	Drawing on existing studies, “life satisfaction (y_1_)” and “future confidence (y_2_)” were used as proxy variables in the questionnaire, and five levels from very dissatisfied to very satisfied were assigned a score of 1–5, respectively.
Core explanatory variables	Participation in the 2012 RSPI	x	Participation = 1, non-participation = 0
Control variables	Year interviewed	Year	Actual year of the questionnaire
	Age	Age	Actual age of survey respondents
	Gender	Gen	Male = 1, Female = 0
	Political Appearance	Par	Member of the CPC = 1, Non-members of the CPC = 0
	Marital status	Mar	With spouse (first marriage, remarriage, cohabitation) = 1, without spouse (unmarried, divorced, widowed) = 0
	Self-assessment of health status	Hea	Assign a value of 1–5 from very healthy to unhealthy, respectively
	Self-assessment of social status	Soc	Assign points 1–5 from low to high, respectively
	Self-assessed income level	Inc	Assign points 1–5 from low to high, respectively
	Region	Code	East = 1, Central = 2, West = 3

#### 4.2.1 Explanatory variables: Farmers’ perception of subjective well-being

Based on existing literature and academic consensus, the CFPS questionnaire used two scales, one of “satisfaction with one’s life” and the other on “confidence in one’s future,” to construct a double-latitude alternative indicator that better meets the needs of the study. The answers to the two questions were rated from very low to very high on a scale of 1–5. The respondents were asked to make subjective evaluations, with mean values of 3.73 and 4.05, between average and high, showing that the subjective well-being of the sample residents was generally at a medium to a high level, with standard deviations of 1.07 and 1.01, confirming the established findings (Yu and Cai, 2019), reflecting the rationality of the indicator construction. The possible reason is that people’s expectations of the future are better than their current feelings, which deserve further attention and exploration.

#### 4.2.2 Core explanatory variables

Specific participation behavior was set as the core explanatory variable, in which participation was assigned a value of 1 and non-participation was assigned a value of 0. Individual residents who also participated in other pension insurance were removed to better reduce possible interference. At the same time, in terms of the actual participation of rural residents, data from 1,067 sample participants who chose to take part in urban residential insurance in 2016 and 2018 were included together to ensure the objectivity and validity of the sample to the greatest extent.

#### 4.2.3 Control variables

Based on previous theoretical analysis, the following control variables (Table 2) were introduced in combination with the indicator portrayal of the questionnaire, controlling as much as possible for the base period variables that affect both the decision to participate and the individual’s subjective well-being.

**TABLE 2:** Descriptive statistic results of variables.

Variable	Sample size	Mean	Standard deviation	Minimum value	Maximum value
Life satisfaction	43,700	3.7269	1.0685	1	5
Future confidence	43,700	4.0527	1.0137	1	5
Enrollment behavior	43,700	0.5476	0.4977	0	1
Gender (male = 1)	43,700	0.4971	0.5000	0	1
Marital status	43,700	0.8345	0.3717	0	1
Political status	43,700	0.0306	0.1722	0	1
Year interviewed	43,700	2015.9	1.6584	2012	2018
Region	43,700	1.9764	0.8443	1	3
Age	43,700	42.0565	13.5185	16	94
Self-assessed income level	43,700	2.5808	1.0456	1	5
Self-assessed social status	43,700	2.9018	1.0666	1	5
Self-assessed health status	43,700	0.5246	0.3097	0	1

At the level of individual characteristics, the maximum age of respondents was 94 years old, with a mean value of 42.06 years, which confirms the necessity and seriousness of the institutional social pension problem compared with the average life expectancy of 77 years. The mean value of gender was 0.497, indicating that the ratio of men to women among respondents was balanced. This is in line with the principle of variable control; political outlook and marital status, which were set as binary dummy variables, with 3% of PPC members in rural areas. The mean value of marital status was 0.83, which is more in line with the actual situation. At the level of social characteristics, the mean values of self-rated income and self-rated social status were 2.58 and 2.90, respectively, which are between low and average in the lower middle range, but the mean value of self-rated health status was 0.52, which is between relatively healthy and very healthy in the upper middle range, indicating that although most rural residents have an average living standard, they are more optimistic about their self-health status, which may be one of the important factors affecting the subjective well-being of rural residents. Therefore, we set it as one of the mediating variables, as tested and discussed later in this article.

### 4.3 Method

Given that the factors affecting individual well-being include many aspects and that well-being is an ordered response variable from 1 to 5, the Order Logistic model was used for regression estimation and its baseline model was set as follows:


(2) $$\text{happinessit}=\alpha_{0}+\beta_{0}\,{\text{X}}_{\text{it}}+\beta_{1}\,{\text{X}}_{\text{it}}^{{}^{\prime }}+\gamma_{1\,t}+\varepsilon_{1\,i\,t}$$

The explanatory variable happinessit in Equation 2 represents the subjective perception of happiness of individual i at time t and is composed of y_1_ and y_2_ together. Xit is the core explanatory variable, which mainly measures the participation of individual i at time t. It is a binary dummy variable, with Xit = 1 indicating participation and vice versa;X′it denotes a series of control variables, including individual characteristics, social characteristics, regional characteristics, etc.; t” γ1t is the time effect, β0 and β1 are the corresponding coefficient matrices, ε1it denotes the random disturbance term.


(3) $${\text{happiness}}_{\text{it}}={\text{b}}_{0}+{\text{b}}_{0}\,{\text{X}}_{\text{it}}+{\text{b}}_{1}\,{\text{X}}_{\text{it}}\times {\text{C}}_{\text{it}}+{\text{b}}_{2}\,{\text{X}}_{\text{it}}^{{}^{\prime }}+\gamma_{2\,t}+\varepsilon_{2\,i\,t}$$

where Cit denotes a series of control variables for individual i at time t. The rest is the same as Equation 2. From the established studies and the current situation in rural areas, the mediating effect of insurance participation behavior on farmers’ subjective well-being is likely to be influenced through self-rated health status and self-rated social status. We attempted to build a mediating effect model to test the original hypothesis.


(4) $$\left\{ \begin{array}{c} {\text{Z}}_{\text{ij}}={\text{d}}_{0}+{\text{d}}_{1}\,{\text{X}}_{\text{it}}+{\text{d}}_{2}\,{\text{S}}_{1}+\gamma_{3\,t}+\varepsilon_{3\,i\,t}\\ {\text{happiness}}_{\text{it}}={\text{g}}_{0}+{\text{g}}_{1}\,{\text{X}}_{\text{it}}+{\text{g}}_{2}\,{\text{Z}}_{\text{it}}+{\text{g}}_{3}\,{\text{X}}_{\text{it}}^{{}^{\prime }}+\gamma_{3\,t}+\varepsilon_{3\,i\,t} \end{array}\right.$$

In the baseline model with self-rated health status as the mediating variable, Zij is the content of self-rated health status, and S1 contains all the variables in the control variables in Equation 2 except for self-rated health status; while in the baseline model with self-rated social status as the mediating variable, Zij is the content of self-rated social status, S1 contains all the variables in the control variables in Equation 1 except for self-rated social status. S1 contains all other variables in the control variables in Equation 1 except for self-rated social status.

## 5. Results

### 5.1 Baseline regression

Based on the OLS benchmark regression, the ordered Order Probit model was used for in-depth estimation, which helped to judge the robustness of the regression results while more clearly reflecting the degree of influence of various factors. For the convenience of explanation, Table 3 reports the results of the benchmark regressions of the two models separately.

**TABLE 3:** Results of the effect of social insurance on farmers’ perception of subjective well-being.^a^

Estimation method	OLS returns	Order Probit	OLS returns	Order Probit
Variable model	y1 (M1)	y1 (M2)	y2 (M3)	y2 (M4)
Participation behavior	0.0498***	0.0486***	0.0580***	0.0588***
	(0.0101)	(0.0107)	(0.0098)	(0.0110)
Year interviewed	0.0264***	0.0309***	0.0022	0.0034
	(0.0029)	(0.0031)	(0.0028)	(0.0032)
Age	0.0033***	0.0041***	−0.0100***	−0.0101***
	(0.0004)	(0.0004)	(0.0004)	(0.0004)
Gender	−0.1050***	−0.1144***	0.0316***	0.0362***
	(0.0097)	(0.0104)	(0.0094)	(0.0107)
Political appearance	0.0393	0.0349	0.0689***	0.0676**
	(0.0275)	(0.0300)	(0.0260)	(0.0310)
Marital status	0.0851***	0.0842***	0.1537***	0.1696***
	(0.0142)	(0.0147)	(0.0138)	(0.0151)
Self-assessed income	0.3122***	0.3400***	0.2324***	0.2631***
	(0.0051)	(0.0052)	(0.0049)	(0.0053)
Region	−0.0242***	−0.0266***	−0.0373***	−0.0423***
	(0.0058)	(0.0062)	(0.0056)	(0.0063)
Constant term	−50.5190***		–0.5872	
	(5.7822)		(5.6191)	
Observations	43,700	43,700	43,700	43,700
*R* ^2^	0.1072		0.0746	

^a^As proxy variables, y1 refers to life satisfaction and y2 refers to future confidence. ** and ***Denote 5% and 1% significance levels, and the standard errors of the estimated coefficients are indicated in parentheses in the model, as below.

1. Overall, the direction and significance of the estimation results of the main explanatory variables of the two models were fully consistent, all passing the 1% test, and the significance of the key variables increased. On the one hand, this shows strong robustness, and on the other hand, it highlights the high applicability of Order Probit regression, indicating that the participation behavior of social insurance is an important factor that influences farmers’ subjective well-being, which is in line with expectations. Participation in social insurance not only significantly improves farmers’ satisfaction with their present life, but also makes farmers’ confidence in the future significantly higher and much higher, realizing the policy expectation of social insurance, which needs to be further consolidated and optimized.
2. In terms of specific variables, the regression results of the control variables are consistent with the findings of existing studies. Marital status and self-rated income are statistically significant at 1%, which is consistent with the common sense judgment. The gender variable showed a negative correlation with current life satisfaction and was significant at 1% statistical significance; however, in terms of confidence in the future, only the age variable showed a significant negative correlation at 1% statistical significance, while gender showed a significant positive correlation. This indicates that the subjective well-being of individual farmers shows a decreasing trend with age. Women had higher results than men in terms of their perception of subjective well-being, but male participants showed higher results in terms of their confidence in the future. Of particular interest is that here, except for the political outlook variable, all other characteristic variables of individuals have a lower degree of confidence in the future than in the current period of satisfaction. A possible explanation for this is that party members bring a capital element or status identity to individual rural residents, making them more confident in the future development of the party and the country.

### 5.2 Individual effects test and endogeneity discussion

#### 5.2.1 Individual effect test

This article uses panel data from the 4-period CFPS, meaning it is necessary to estimate the parameters using the individual effects model and compare them with baseline regression. The Hausman test results show that the chi-square test statistic for dependent variable 1, life satisfaction is 192.27 (*p* = 0.0000), and the chi-square test statistic for dependent variable 2, future confidence is 93.51 (*p* = 0.0000). All dependent variables were highly significant at 1% statistical significance, so fixed effects were used. Further observations revealed that the direction, estimates, and significance of the effect of participation behavior on the two dependent variables were largely consistent, again confirming the robustness of the findings.

#### 5.2.2 Endogeneity discussion

Given the inevitability of the endogeneity problem, the article attempts to dissipate the omission bias phenomenon that may be brought about by the endogeneity problem by controlling for more variables and PSM tests. Since participation behavior is a binary dummy variable, participation behavior was first used as the treatment variable, and covariates including age, gender, political affiliation, marital status, region, and self-rated income were included in the series. Propensity score matching analysis is estimated by the logit model. Results showed a good *R*^2^ of model fit and a high overall significance level. The covariates age, gender, political outlook, marital status, self-assessed income, and region showed positive correlations with participation in social insurance and were statistically significant at 1%. In terms of econometric rigor, Table 4 provides an in-depth calculation of the standard deviations of the treatment and control groups based on all matching variables with a *t*-test.

**TABLE 4:** Matching balance test results.

Variables	Sample	Mean		Marking deviation/(%)	Standard deviation reduction/(%)	*T*-value	*P*-value
		Processing group	Control group				
Age	Before matching	44.126	39.551	33.9	99.4	35.73	0.000***
	After matching	44.126	44.156	–0.2		–0.28	0.783
Gender	Before matching	0.50161	0.49173	2	26	2.06	0.040**
	After matching	0.50161	0.4943	1.5		1.6	0.11
Political Appearance	Before matching	0.03518	0.02499	6	–69.3	6.16	0.000***
	After matching	0.03518	0.01793	10.1		11.76	0.000***
Marital status	Before matching	0.89942	0.75583	38.7	93.8	40.96	0.000***
	After matching	0.89942	0.90828	–2.4		–3.29	0.001***
Self-assessed income	Before matching	2.6254	2.5269	9.4	99.4	9.81	0.000***
	After matching	2.6254	2.6248	0.1		0.07	0.947
Region	Before matching	2.0686	1.8647	24.3	95.1	25.31	0.000***
	After matching	2.0686	2.0587	1.2		1.29	0.196

** and ***Denote 5% and 1% significance levels.

**FIGURE 2:**
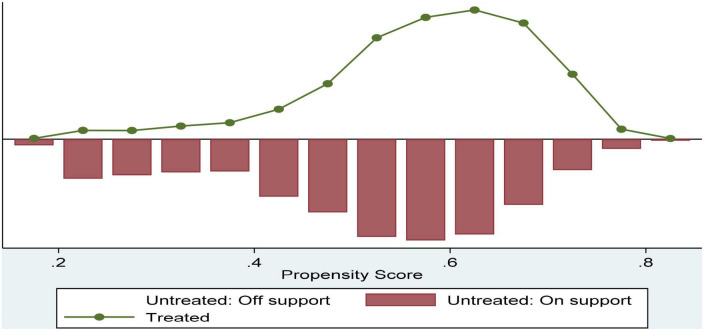
Common range of values for propensity score.

The results show that the control range of the standard deviation of the six variables is significantly lower. The absolute value of the standardized deviation of all variables after matching was less than 10, except for political outlook, which is 10.1%, and the overall standardized deviation of variables is also less than 15%, which is within a perfectly acceptable range. Moreover, the *t*-test results do not reject the original hypothesis that there are no systematic differences except for political outlook and marital status. The *t*-test results also did not reject the original hypothesis of no systematic differences, except for political appearance and marital status, again indicating the reliability of the propensity value matching estimates. Figure 2 visualizes the distribution characteristics of the absolute values of bias before and after matching, indicating that the treatment and control groups are highly similar and fully meet the needs of the study.

### 5.3 Robustness tests and extended analysis

#### 5.3.1 Robustness tests

This study also conducted a double test. First, OLS and Order Probit were used to examine the effect of participating in social insurance and how this behavior affects farmers’ perceptions of their subjective well-being, respectively. The results, although slightly different in value, were approximately the same in direction and significance, indicating that the conclusions were robust. On the other hand, to eliminate other effects that may arise from the extreme values of the sample, the robustness test was conducted again using the Winsorization data processing method. The results, shown in Table 5, were robust overall and some of the estimated coefficients even become more significant.

**TABLE 5:** Robustness test results.

	Life satisfaction	Future confidence level
	
	y1 (Order Probit)	y2 (Order Probit)
Winsorization processing Enrollment after processing	0.0486***	0.0588***
	(0.0107)	(0.0110)
Year of enrollment	0.0309***	0.0034
	(0.0031)	(0.0032)
Age	0.0041***	−0.0101***
	(0.0004)	(0.0004)
Gender	−0.1144***	0.0362***
	(0.0104)	(0.0107)
Political appearance	0.0349	0.0676**
	(0.0300)	(0.0310)
Marital status	0.0842***	0.1696***
	(0.0147)	(0.0151)
Self-assessed income	0.3400***	0.2631***
	(0.0052)	(0.0053)
Regional distribution	−0.0266***	−0.0423***
	(0.0062)	(0.0063)
Observations	43,700	43,700

** and ***Denote 5% and 1% significance levels.

In addition, this study further tested the dependent variable with a one-period lag by changing the instrumental variables and the results were shown to be robust and valid. Due to limitations in article length, the reported results are not repeated in detail here.

#### 5.3.2 Expanded analysis

The above analysis of the effect of social insurance participation on farmers’ perception of subjective well-being under the full sample were obtained as the average effect of participation on all farmers’ subjectivity perceptions, and did not consider the heterogeneous effect of participation behavior on farmers’ perception of subjective well-being. The heterogeneous effects of social insurance participation behavior are reported in Table 6.

**TABLE 6:** Heterogeneous effects of social insurance participation on farmers’ perception of subjective well-being: sub-sample regression results.

Variables	Male	Female	Eastern Region	Central Region	Western Region	Under 45 years of age	45–60 years old	Over 60 years old
	Life satisfaction	Life satisfaction	Life satisfaction	Life satisfaction	Life satisfaction	Life satisfaction	Life satisfaction	Life satisfaction
Participation behavior	0.0727***	0.0727***	0.113***	–0.00533	0.107***	−0.00199**	0.00507***	0.0134***
	–0.0147	–0.0146	–0.017	–0.0195	–0.018	–0.0009	–0.0004	–0.0034
Self-assessed income	0.395***	0.306***	0.361***	0.351***	0.326***	0.334***	0.344***	0.312***
	–0.0089	–0.0078	–0.0098	–0.011	–0.0098	–0.0082	–0.0059	–0.0186
Control variables	Yes	Yes	Yes	Yes	Yes	Yes	Yes	Yes
Sample observation size	21,725	21,975	16,104	12,525	15,071	24,597	43,700	3,024

**Variables**	**Male**	**Female**	**Eastern region**	**Central region**	**Western region**	**Under 45 years of age**	**45–60 years old**	**Over 60 years old**
	**Future confidence level**	**Future confidence level**	**Future confidence level**	**Future confidence level**	**Future confidence level**	**Future confidence level**	**Future confidence level**	**Future confidence level**

Participation behavior	0.0143	0.0434***	0.0582***	–0.0296	0.0639***	−0.00686***	−0.00857***	–0.00111
	–0.0151	–0.0149	–0.0175	–0.02	–0.0183	–0.001	–0.0004	–0.0033
Self-assessed income	0.280***	0.246***	0.272***	0.276***	0.245***	0.221***	0.265***	0.326***
	–0.0086	–0.0076	–0.0094	–0.011	–0.0094	–0.0081	–0.0057	–0.0183
Control variables	Yes	Yes	Yes	Yes	Yes	Yes	Yes	Yes
Sample observation size	21,725	21,975	16,104	12,525	15,071	23,391	43,700	3,024

** and ***Denote 5% and 1% significance levels.

1. *Effect of gender.* Columns 1 and 2 of Table 7 report on the different perceptions of insured farmers on subjective well-being according to their gender. In general, the difference in life satisfaction between insured men and women was not significant. A comparison of the regression coefficients of the self-rated incomes of male and female participants reveals the positive effect of self-rated income on life satisfaction and the degree of confidence they have in their future, which was higher for men than for women. Compared with men, women’s confidence in the future is significantly higher. The reason for this may be related to the traditional gender concept that women have relatively fewer negative emotions.

**TABLE 7:** Table of results of Sobel’s mediating effect test.

Variables	Intermediary effect regression results				Intermediary effect regression results			
	Life satisfaction(3)(9)				Future confidence level(6)(12)			
	Regression coefficient	Estimated standard error	*T*-value	*P*-value	Regression coefficient	Estimated standard error	*T*-value	*P*-value
Participation behavior	0.0578	0.0099	5.83	0.000	0.0681	0.0095	7.14	0.000
Age	0.0077	0.0004	19.74	0.000	–0.0055	0.0004	–14.6	0.000
Gender	–0.1474	0.0097	–15.23	0.000	–0.0114	0.0093	–1.23	0.220
Political appearance	0.0146	0.0278	0.52	0.601	0.0575	0.0268	2.15	0.032
Marital status	0.0773	0.0137	5.64	0.000	0.1492	0.0132	11.32	0.000
Self-assessed income	0.2944	0.0046	63.82	0.000	0.2084	0.0044	46.94	0.000
Region	–0.0133	0.0057	–2.33	0.020	–0.0263	0.0055	–4.80	0.000
Constant term	2.3303	0.0266	87.49	0.000	3.3102	0.0256	129.2	0.000
Sample size		43,700				43,700		
*R* ^2^		0.1318				0.1067		
*F*-value		828.82				652.41		
Self-assessment of health status(1–6)	0.0599	0.0165	36.32	0.000	0.6290	0.0159	39.64	0.000
Sobel *Z* value		–5.717				–5.706		
Intermediary effect		–0.1683				–0.1500		
Intermediary effect as a percentage (%)		–20.24%				–17.65%		
Participation behavior	0.0330	0.0098	3.36	0.001	0.0451	0.0095	4.73	0.000
Age	0.0008	0.0004	2.15	0.032	–0.0122	0.0004	–33.7	0.000
Gender	–0.0832	0.0095	–8.73	0.000	0.0511	0.0093	5.53	0.000
Political appearance	–0.0303	0.0276	–1.10	0.272	0.0215	0.0268	0.80	0.422
Marital status	0.0797	0.0136	5.88	0.000	0.1521	0.0132	11.55	0.000
Self-assessed income	0.1989	0.0052	38.32	0.000	0.1323	0.0050	26.28	0.000
Region	–0.0387	0.0057	–6.85	0.020	–0.0450	0.0055	–9.11	0.000
Constant term	2.5130	0.0242	104.05	0.000	3.5542	0.0234	151.6	0.000
Sample size		43,700				43,700		
*R* ^2^		0.1482				0.1085		
*F*-value		950.45				664.62		
Self-assessment of social status(7–12)	0.2416	0.0052	46.79	0.000	0.2043	0.0050	40.77	0.000
Sobel *Z* value		6.781				6.758		
Intermediary effect		0.4564				0.2822		
Intermediary effect as a percentage(%)		31.34%				22.01%

2. *Effect of region.* Columns 3–5 of Table 7 show the regression results relating to the subjective perception of well-being of insured farmers in the east, central and west regions of China. An increase in self-assessed income had a significant positive effect on both life satisfaction and the degree of future confidence experienced by insured farmers. Further observation indicates that participation in the insurance system significantly increased the perception of subjective happiness in farmers in the east and west regions, with the former having greater life satisfaction than the latter; while the opposite is true for the degree of future confidence, and the opposite effect observed for both central regions. This might be because there are significant differences in the implementation of social insurance between regions, and different regions have different degrees of openness to adopting the policy. For example, the eastern region has a better level of economic development and a higher degree of acceptance of social insurance policies, and there are relatively fewer pressures on residents to pay contributions, meaning farmers in this region have higher current life satisfaction with social insurance implementation. By contrast, farmers in the western region need to overcome real economic pressures to participate in the policy, hoping that the future pension will provide a stable source of income and livelihood security.
3. *Effects by age group.* Considering the realities of rural life, columns 6–8 of Table 7 show the respective regression results relating to the life satisfaction and confidence in the future of insured farmers, divided into three age groups, including those below 45 years old, people aged 45–60 years old, and those above 60 years old. For all age groups, an increase in self-rated income showed a significant positive correlation with an increase in subjective well-being. In terms of future confidence, all age groups showed a negative correlation, indicating that the existing policy needs to be publicly strengthened and that farmers generally have doubts about longer-term policy expectations. In terms of current life satisfaction, those under 45 years old show a significant negative correlation, indicating that participating farmers have an overall distrust of whether the “new rural insurance” policy can guarantee their future retirement for a longer period of time. Those over 45 years old show a significant positive correlation, and those over 60 years old are significantly higher than those between 45 and 60 years old. This is because, residents who are not covered by the basic pension insurance for urban workers who have reached the age of 60 can receive a certain level of monthly basic pension without paying any fees, and this income will rise. However, for residents under the age of 60, the contribution cycle requires at least 15 years of contributions.

## 6 Discussion

With the help of Sobel’s mediator test, the mediating effect model Equation 3 was validated, and then the mechanism of the mediating effect and the impact effect were explored. Due to the limitation of data availability, this manuscript focuses on whether participation behavior leads to the improvement of farmers’ subjective well-being by enhancing their self-rated health status and self-rated social status. Due to further limitations of space, only the core test results are presented in Table 7.

As the results displayed in Table 7 indicate, the mediating variables showed a significant positive effect, not only because they significantly pass the 1% statistical significance test but also because the Sobel Z statistic was significant, indicating that self-rated health status and self-rated social status play a fully mediating role in the transmission mechanism. The Sobel Z values of self-rated health status and self-rated social status on life satisfaction and future confidence were –5.717, –5.706, 6.781, and 6.758. The proportion of mediating effects were –20.24, –17.65, 31.34, and 22.01%, indicating that the existing mediating effects of self-rated health status and self-rated social status could explain (including masking) the effects of insurance participation on rural residents’ perception of subjective well-being were 20.24, 17.65, 31.34, and 22.01, which matched expectations. As mediating paths, health status and social status have different degrees of influence on current life satisfaction and confidence in the future. The mediating effects of self-rated health status and self-rated social status on current life satisfaction are greater than those on future confidence to a significant extent.

The potential mechanisms of the impact of social insurance on farmers’ subjective well-being are as follows: (1) as one of the economic sources that guarantee farmers’ lives, social insurance pensions have the effect of reducing expected risks. For the insured individuals, whether the pension has been received or not, it is likely to affect their psychological expectation of future life risks and uncertainties, thus obscuring the individuals’ judgment of their health status, subjectively paying more attention to their health status, objectively generating the psychological worry of suffering from loss and gain, and enhancing their subjective sense of well-being. Although the protective function of social insurance hedges some expected life risks, the contributions still create payment pressures for people with poor health conditions, which in turn reduces the positive impact of social insurance on their psychological well-being. For farmers who have already received pensions, since they are not under pressure to pay contributions and have stable livelihood security for the foreseeable future, they have a higher level of pursuit for a long and healthy life, which to a certain extent increases their judgment of their health risks and creates the “Rosenthal effect.” (2) The act of participating in insurance leads them to have a certain perception of their “identity” and this foundation enables them to enjoy the “retirement treatment” of city people. As an important variable of individual identity perception, self-assessed social status expresses the individual’s recognition of their characteristics, which indirectly affects their judgment of their subjective well-being, indicating the positive mediating effect of self-assessed social status.

## 7 Conclusion

This study first used the Order Probit regression model to provide an in-depth analysis of rural residents’ participation in insurance, examining its effect on individual subjective well-being before further exploring its possible effects on the subjective well-being of farmers from different regions, examining the perspectives of people of different genders across various age groups. The study also tested the influence of health status and self-rated social status on the farmers’ subjective well-being and the moderating mechanisms of this perception. All results passed the robustness test, further confirming the reliability of the findings.

This article analyzed the impact of participation in social insurance on the behavior of rural residents and their subjective well-being. The results indicate that first, participation behavior can significantly improve rural residents’ subjective well-being. Second, the heterogeneity study showed that gender differences in life satisfaction among insured farmers were not significant. The positive effects of self-assessed income on life satisfaction and confidence in the future were higher for men than for women; however, men had significantly lower confidence in the future than women. An increase in self-assessed income significantly increases the life satisfaction and future confidence of rural insured residents, but the act of participating in insurance significantly increased farmers’ perception of subjective happiness in the eastern and western regions of China. The eastern region showed greater life satisfaction than the west, but the opposite was true in terms of confidence in the future, with the central region showing a negative effect. Third, an increase in self-assessed income significantly increased farmers’ perception of subjective well-being in all age groups. However, specific to the act of participation, all age groups showed a negative correlation in terms of the degree of confidence they had in the future. Concerning current life satisfaction, people aged below 45 years old showed a significant negative correlation, those above 45 years old showed a significant positive correlation, and those aged over 60 years old had a significantly higher than those between 45 and 60 years old. Fourth, the act of participating in insurance can significantly obscure farmers’ health status ratings and significantly contribute to the improvement of individuals’ self-rated social status, which in turn affects their perception of their subjective happiness. Further tests exploring the mediation mechanism suggested that rural residents with poor health status and good overall conditions benefit most from social insurance.

Based on the above conclusions, the possible policy insights of the present study include: first, that even though the pursuit of a better life is undertaken for different reasons at different points in life and can vary among individuals, during the critical period in which rural revitalization and accelerating common prosperity are being encouraged and promoted, there are still shortcomings in terms of how best to apply them to agriculture and farmers in rural areas. Taking into account the aim to comprehensively eradicate poverty, when addressing the question of how best to further consolidate and deepen these aims and link them with rural revitalization, we need to continue to find ways of improving the living standards of low-income groups. Eliminating the risk of returning to poverty due to illness is an important aspect of promoting common prosperity and a more comprehensive realization of rural revitalization in the future. Second, the construction of the rural pension insurance system still has a long way to go. Traditionally, absolute income has been used to measure the welfare status of rural residents, but there are large limitations to this approach. Absolute income as a flow indicator cannot reflect the differences in the wealth accumulation status of individual households, nor the differences in expenditure burden experienced by different households. Questions remain about how the basic social security functions of the rural basic pension system can be realized and how the basic livelihoods of rural residents can be protected more effectively. When implementing social pension security policies according to local conditions, is also necessary to take into account regional differences, economic differences, and other factors and encourage more comprehensive full coverage of basic pension insurance when appropriate. Thirdly, effectively enhancing the subjective sense of well-being of rural residents is key to promoting rural revitalization strategies. Strong agriculture, beautiful countryside, and rich farmers are the general goal of agricultural and rural modernization and a strategic goal of rural revitalization. Therefore, we need to pay attention to the growth of farmers’ income and the improvement of their living standards and health conditions, which will in turn influence their subjective perception of their happiness. It is also necessary to continuously improve the social identity of rural residents and their social status by increasing basic medical insurance system coverage in rural areas and strengthening the public health service system through construction to effectively promote improvements in rural residents’ subjective sense of well-being, which an important aim of current responses to coping with an aging society.

It should be noted that the empirical analysis undertaken in this study has some shortcomings. The first is that, due to the data limitation of CFPS, we constructed the empirical model based on the binary dummy variable of whether or not people participate in the insurance system. It is important to note that there are differences in the amount paid and types of insurance received and that changes in people’s perceptions before and after participation in these systems are not reflected in the available data. There are also differences in post-retirement benefits due to the payment of different insurance premiums, which bring different degrees of impact on happiness that require further study.

Second, due to limitations in methods and data, further optimization should be undertaken to address and solve problems of reciprocal causality. In addition, whether the effect of social insurance on farmers’ subjective well-being changes over time is also a topic worth further study. Finally, it is difficult to explore the influence of more control variables on the article’s findings because there are too many missing values in individual and household information in the sample data. These questions have yet to be discussed, tested, and answered by relevant follow-up studies. Further attention should be paid to how to address these limitations in order to apply a more developed model more generally in the assessment of whether local welfare policy is adequate and an indicator system for monitoring the progress of well-being, etc., should be established. Further research is important for the continuous implementation, improvement, and promotion of social security policies in the future, thus contributing to the realization of common prosperity.

## 8 Data availability statement

This data can be found here: The data underlying the results presented in the study are available from CFPS database. http://www.isss.pku.edu.cn/cfps/.

## 9 Author contributions

YC: conceptualization, methodology, software, and writing – reviewing and editing. DZ: supervision. Both authors contributed to the article and approved the submitted version.

## Footnotes

1 The original questionnaire options were: 1 = very healthy, 2 = very healthy, 3 = fairly healthy, 4 = fair (no readout), 5 = unhealthy. To unify the positive indicators, the data were reversed to 1 = very healthy, 0.75 = very healthy, 0.5 = comparatively healthy, 0.25 = average, and 0 = unhealthy. In terms of regional distribution, according to the National Bureau of Statistics, the eastern, central and western parts of China are specifically covered as follows: the eastern region includes Beijing, Tianjin, Hebei, Liaoning, Shanghai, Jiangsu, Zhejiang, Fujian, Shandong, and Guangdong; the western region includes Chongqing, Sichuan, Guizhou, Yunnan, Shaanxi, Gansu, Guangxi, Inner Mongolia, and Xinjiang; the central region includes Shanxi, Jilin, Heilongjiang, Anhui, Jiangxi, Henan, Hubei, and Hunan.
